# Conducting Patient-Pathway Analysis to Inform Programming of Tuberculosis Services: Methods

**DOI:** 10.1093/infdis/jix387

**Published:** 2017-11-06

**Authors:** Christy L Hanson, Mike Osberg, Jessie Brown, George Durham, Daniel P Chin

**Affiliations:** 1 Macalester College, St. Paul, Minnesota; 2 Bill and Melinda Gates Foundation and; 3 Linksbridge, Seattle, Washington

**Keywords:** tuberculosis, patient pathway analysis, care seeking, diagnosis, private sector

## Abstract

Patient-centered care is a central pillar of the World Health Organization’s End TB Strategy. Understanding where patients access health services is a first step to planning for the placement of services to meet patient needs and preferences. The patient-pathway analysis (PPA) methodology detailed in this article was developed to better understand the alignment between patient care seeking and tuberculosis service availability. A PPA describes the steps that people with tuberculosis take from the initial care visit to cure. The results of a PPA reveal programmatic gaps in care seeking, diagnosis, treatment initiation, and continuity of care. They can be used as inputs to an evidence-based process of identifying and developing interventions to address the gaps in patient care. This paper summarizes the steps to conduct a PPA and serves as the basis for understanding country case studies that profile the use of PPA.

According to the World Health Organization (WHO), >40% of the >10 million people with incident tuberculosis in 2015 were not notified to national tuberculosis control programs (NTPs) [1]. Whether these people received appropriate diagnosis and treatment or not is unknown. Findings from the Demographic and Health Surveys Program suggest that these so-called missing cases fall into 3 groups of patients [2]. First, some patients had not yet sought care. They may not have had severe symptoms, or they delayed care seeking because of financial, geographic, or other barriers to accessing care. Among the patients who had sought care, some accessed the private (ie, nonstate) sector. They may not have received diagnostic confirmation of tuberculosis, or they might have received a diagnosis and treatment but were not notified to the NTP. Third, among the patients who sought care in the public sector, some may have received a diagnosis and treatment but were not notified to the NTP.

Obviously, finding these missing cases is important because all people with tuberculosis deserve to receive appropriate care. In addition, those who do not access care and those who receive a diagnosis and are treated incorrectly continue to transmit tuberculosis in the community. Many studies have shown that patients treated in the private sector frequently do not receive proper tuberculosis testing or drug treatment, and those who initiate treatment frequently do not finish [3, 4]. Therefore, these cases may also be an important source of drug-resistant tuberculosis.

Patient-centered care is a core principle of the WHO End TB Strategy [5]. However, tuberculosis prevention and care services are often planned from the top down, primarily on the basis of available capacities of the health system. Seldom do tuberculosis programs consider where services should be positioned to meet patients where they are. Instead of thinking about where they can provide services, programs should consider where they should position services, to most efficiently meet patients with tuberculosis where they are.

The patient-pathway analysis (PPA) methodology was developed to better understand the alignment between patient care seeking and tuberculosis service availability. The PPA builds on previous methods of identifying gaps in a tuberculosis program. The onion model, commonly used by the WHO, identifies important places where a patient may drop out of care on their way to achieving cure. Similarly, the care cascade model, often used by the human immunodeficiency virus care community and recently applied to tuberculosis programs, attempts to calculate the frequency of dropout along the pathway to care. The PPA is distinct from and complements care cascade analyses in several ways. First, data on care seeking and service availability are typically more readily available than data on diagnosis and treatment of tuberculosis in nonnotified patients (which, if not available, must be estimated in care cascade studies). Second, the PPA can provide data points that can inform care cascade estimates. Finally, the PPA can provide insight as to why patients drop out at points along the cascade, by highlighting misalignment between patient care seeking and service availability.

The PPA aims to describe the steps patients with tuberculosis take from the initial point of seeking care to the point of achieving cure. At the same time, the analysis reviews the availability of tuberculosis screening, diagnosis, and treatment at various levels of the health system. By examining the alignment of care seeking with service availability, the PPA may reveal where patients with tuberculosis experience delay during care seeking or treatment initiation, access inappropriate care, or are lost to follow-up during their journey toward cure.

The intent of the PPA is to help national tuberculosis programs more accurately identify some of the health system alignment gaps that can be addressed through targeted program interventions. Equipped with data from a PPA, tuberculosis programs can plan tuberculosis prevention and care services in a manner that responds to patient care-seeking preferences and options.

## DATA SOURCES

### Care-Seeking Data for Patients With Tuberculosis

To complete a PPA, 2 basic types of information are needed: patient care-seeking patterns and tuberculosis service availability. For patient care-seeking patterns, it is ideal to capture where in the health system patients initiate care when they have tuberculosis symptoms, because programs should aim to provide appropriate care as soon as patients enter the health system. One of the best sources of tuberculosis-specific care-seeking data is a tuberculosis prevalence survey. While a tuberculosis prevalence survey provides tuberculosis-specific care-seeking data, the sample size of patients confirmed to have tuberculosis is commonly small and will not allow for robust subnational analysis. To consider a larger sample, one can also use data from other population-based surveys that capture care seeking for general, respiratory, or other illnesses. Many countries use health utilization surveys or Demographic and Health Surveys that reflect this information [2]. These indicators have been found to serve as reasonable proxies for tuberculosis-specific care seeking [6, 7]. Where both types of data are available, one can triangulate the sources to optimize the sample size and indicator selection.

### Data on Availability of Tuberculosis Services

For information on the availability of tuberculosis services, one can use nationally representative surveys of service availability in the healthcare network. Two key sources are Service Provision Assessment surveys, which are part of the Demographic and Health Surveys suite, and the Service Availability Readiness Assessment, coordinated by the WHO. If these surveys are not available, service availability can be determined from national health facility inventories and NTP registries of health facilities with tuberculosis diagnostic capacity and/or treatment capacity.

Tuberculosis services for which data could be available include smear microscopy, capacity for performing molecular tests such as Xpert (on site or via referral to another site), radiography, specimen transport, patient referral, and first-line antituberculosis drugs. Data may also be available to assess the availability of patient support, treatment adherence support, conventional drug-susceptibility testing, and drugs for multidrug-resistant tuberculosis.

### Assigning Health Facility Categories

When reviewing data on care seeking and service availability, it is important to know that data from different sources may not use the same naming conventions for categorizing facility levels or types. Therefore, we used a standardized naming convention to organize the data. For each data source, facilities were categorized into health system levels, health facility type, and sector type. The 2 primary sectors used in the PPA are the public sector and the private sector. In many countries, it is useful to split private-sector facilities into formal private facilities and informal private facilities, where the informal private facilities provide access to practitioners with little or no formal health training (eg, individuals at drug shops and traditional healers). The categorization of nongovernmental organizations into the public or private sector should align with the country’s norms for categorization. Categorization may differ by country, reflecting the diversity of health systems.

The standard health facility levels used by the PPA are as follows. Level 0 (L0) refers to basic, community-based care. L0 services include basic triage, provision of health information, and essential prevention and care. Services are commonly provided as an extension of facility-based care and are provided by volunteers or paramedical staff with limited formal training. Specimen capture may be available, and L0 staff may serve as treatment supporters for patients with tuberculosis. Examples include community health workers (public) and traditional healers, mobile clinics, voluntary counseling and testing service facilities (private). Level 1 (L1) facilities provide primary healthcare. L1 services are commonly provided on an outpatient basis, by nurses, midwives, or private physicians. Some basic diagnostic services, including basic microscopy, and essential medicines may be available. Rural health centers and private clinics are examples in public and private sectors, respectively. Level 2 (L2) facilities provide primary healthcare, as well as more-advanced care. L2 facilities commonly have more-extensive diagnostic and treatment options and can provide both outpatient and inpatient care. Examples include district hospitals (public) and rural or nongovernmental organization–affiliated private hospitals (private). Level 3 (L3) facilities provide specialized care and have a large inpatient capacity. L3 facilities provide access to specialized physicians and have more-sophisticated diagnostic and treatment capabilities. Examples include referral or teaching hospitals (public) and urban private hospitals (private).

## DATA ANALYSIS

To estimate the likelihood that patients will receive tuberculosis diagnostic and treatment services at the facility where they initiate care, we can analyze the most-common types of health facilities for care initiation and the capacity of these facilities to provide tuberculosis diagnostic and treatment services (Figure 2). The PPA can be conducted at the national and subnational levels. The analysis can also be performed at an intermediate level (eg, urban vs rural areas). Below are 7 indicators commonly used in the PPA. These indictors refer to columns included in Figure 2. This figure was generated using fictitious data for demonstration purposes.

**Figure 1.**
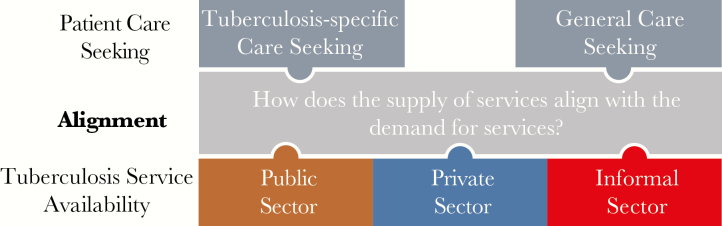
Assessing alignment of patient care seeking and health systems. An analysis of the alignment of tuberculosis patient care-seeking and health service delivery can help programs to identify gaps that can be closed through prioritized and targeted planning. The assessment results should be considered one input into a process of better understanding your tuberculosis epidemic and planning based on evidence.

**Figure 2.**
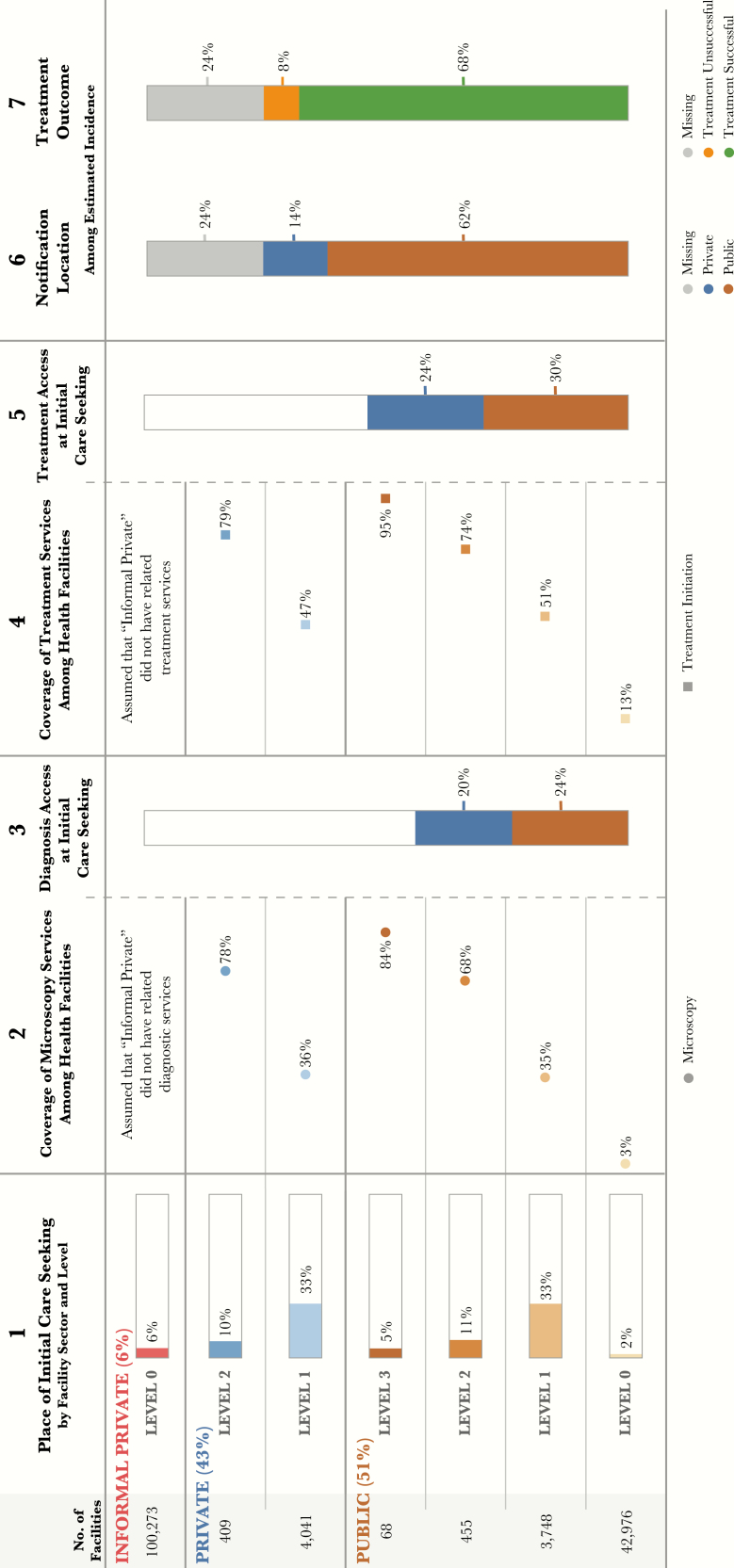
Demo patient-pathway visual. The patient-pathway analysis visual describes several potential steps along a patient’s journey for tuberculosis care. The first column shows where patients initiate their care-seeking journey across different sectors and levels of the healthcare system. Column 2 estimates the percentage of health facilities at each level and sector that have microscopy available in the facility. Column 3 estimates the likelihood that a patient will start their care-seeking journey at a facility that has diagnostic tools available. This is calculated by multiplying the share of patients who seek care (column 1) at each sector and level by the microscopy coverage. The fourth column shows the coverage of tuberculosis treatment services at each sector and level of the health system. Similar to column 3, column 5 estimates the likelihood of a patient starting their care-seeking journey in a facility that has tuberculosis treatment services available. This is calculated by multiplying care seeking at each sector and level (column 1) by the coverage of tuberculosis treatment services (column 4). The final 2 columns show the location of notification and treatment outcomes of notified patients as a share of the overall tuberculosis burden.

### Initial Care Seeking

“Initial care seeking” denotes the proportion of patients who initiate care, by facility level and sector. The numerator is the number of patients initiating care for illness or symptoms (preferably tuberculosis symptoms) in a specific facility level and sector. The denominator is the total number of patients initiating care for illness or symptoms (preferably tuberculosis symptoms) in the survey population. In Figure 2, 34% of people with tuberculosis symptoms initiate care in the formal private sector, and 10% initiate care at one of the 409 private L2 facilities.

### Diagnostic Coverage

“Diagnostic coverage” denotes the proportion of health facilities with tuberculosis diagnostic services, by facility level and sector. The numerator is the number of health facilities with tuberculosis diagnostic services, by facility level and sector. This can be calculated for each type of tuberculosis diagnostic service (eg, smear microscopy, Xpert analysis, and radiography). The denominator is the total number of health facilities, by facility level and sector.

In Figure 2, among the 409 private L2 facilities, 78% have microscopy. An identical data point could be reported for radiography, Xpert, or other diagnostic tools. Alternatively, the percentage of facilities with any type of tuberculosis diagnostic service could be reported.

### Diagnostic Access

“Diagnostic access at initial care seeking” denotes the proportion of patients who initiate care in a facility with tuberculosis diagnostic services, by facility level and sector. This indicator is the product of initial care seeking and diagnostic coverage. This indicator can be interpreted as the likelihood that a patient has access to tuberculosis diagnostic services at their first visit to the healthcare system. One can sum diagnostic access for all health facilities and sectors to derive an indicator of overall diagnostic access. This indicator can be calculated for each type of tuberculosis diagnostic services or for a combination of diagnostic tools of interest.

In Figure 2, 10% of care seekers seek care in private-sector L2 facilities. For those 10%, the likelihood of accessing a facility with microscopy is 78%. Thus, an estimated 7.8% of all care seekers access microscopy at a private L2 facility. Access to microscopy for the entire formal private-sector (L1 and L2 combined) is calculated as diagnostic access in L2 facilities [10% × 78%] plus diagnostic access in L1 facilities [33% × 36%], resulting in 20% of all care seekers accessing a private-sector facility with microscopy at initial care seeking. Thus, 44% of care seekers (20% in the private sector plus 24% in the public sector) access a facility with microscopy at their initial visit to the healthcare system.

### Treatment Coverage

“Treatment coverage” denotes the proportion of health facilities that have antituberculosis medicines or that can supervise patients during treatment, by facility level and sector. The numerator used to calculate treatment coverage is the number of facilities that have antituberculosis drugs or that can supervise patients during treatment, by facility level and sector. The denominator is the total number of health facilities, by facility level and sector. In Figure 2, among the 4041 private L1 facilities, 47% offer tuberculosis treatment services.

### Treatment Access

“Treatment access at initial care seeking” denotes the proportion of patients who initiate care in a facility that has antituberculosis medicines or that can supervise patients during treatment, by facility level and sector. This indicator is the product of care seeking and treatment coverage. This indicator can be interpreted as the likelihood that a patient has access to tuberculosis treatment services on their first visit to the healthcare system. One can sum the treatment access for all health facilities and sectors to derive an indicator of overall treatment access. This indicator can be calculated for antituberculosis drugs and/or treatment supervision.

In Figure 2, access to treatment in the private sector is calculated by multiplying the proportion of care seeking at each level by the coverage of treatment services at that level, such that treatment access in L1 facilities [10% × 79%] plus that in L2 facilities [33% × 47%] results in 24% of all care seekers accessing a private-sector facility with tuberculosis treatment services at their initial visit. Thus, 54% of care seekers (24% in the private sector and 30% in the public sector) access a facility with tuberculosis treatment at their initial visit to the healthcare system.

### Notification Location

“Notification location” denotes the location of case notification for cases notified to the WHO. This indicator is calculated using the total estimated burden for the 5 countries profiled, and nonnotified cases are labeled as “missing.” In Figure 2, 14% of all estimated incident cases are notified from the private sector, corresponding to 18% of all notifications.

### Treatment Outcome

“Treatment outcomes” denote the treatment outcomes among cases notified to the WHO. Treatment success rates from the 2016 WHO report for each country are used with country-specific notified cases to calculate the number of patients who successfully completed treatment and those who did not successfully complete treatment. These numbers are then used to calculate the total estimated burden for the 5 countries profiled, with nonnotified cases labeled as “missing.” In Figure 2, 8% of all estimated incident cases are notified and have an unsuccessful treatment outcome; 11% of notified cases have an unsuccessful treatment outcome.

## DATA VISUALIZATION

Data visualization is an important component of the PPA because it provides an easy-to-understand summary of the alignment between care seeking and service availability at various levels of the health system. The visualization allows the reader to move through each step of the pathway and see how patients may encounter challenges as they visit different health facilities. In Figure 2, each column of the standard visualization corresponds to one of the 5 indicators described above.

There are several software packages with data visualization capabilities. In this supplement, final visualizations of the pathways are created in Adobe Illustrator, but Tableau is the primary software used to complete the analysis and drive the underlying PPA visualizations. An example of how PPA is presented in Tableau is available at: https://public.tableau.com/profile/mike.osberg1526#!/vizhome/DemoPPA/FullPPA. The example contains an interactive PPA built in Tableau, using fictional data of the type that might be used in a PPA. The Tableau workbook is free to download and provides users the necessary underlying structure to design a PPA in Tableau (https://www.tableau.com, accessed 1 May 2017). Users can also download the source Excel sheet once they have downloaded the Tableau workbook. The source Excel sheet provides an example of how data for a PPA should be structured.

## INTERPRETATION

The PPA is a tool to determine the efficacy of tuberculosis service delivery to patients. The interpretation of results from a PPA can be used as part of an evidence-based process to identify, prioritize, and address the gaps in tuberculosis diagnosis and treatment. When interpreting PPA results, it is important to consider the context of the tuberculosis program (eg, the diagnostic and treatment algorithms and locations planned by the NTP and the availability of other data not part of the PPA). Any interpretations of PPA results should be discussed and confirmed with country experts. The visualization of PPA data helps with data interpretation (Figure 2). The discussion below describes how the basic PPA indicators are commonly used and interpreted.

### Initial Care Seeking

Patients tend to initiate care at health facilities in locations and with types of providers that they prefer. The varied distribution of care initiation across different types of health facilities is due to the availability of providers and the acceptability and affordability of providers to patients. The distribution of initial care seeking can be compared to the distribution of the source of tuberculosis notification to determine whether there is significant mismatch. For example, if 50% of care initiations are in the private sector but only 10% of case notifications come from the private sector, there may be significant dropout along the care-seeking pathway after care initiation in the private sector. Alternatively, a significant proportion of these patients may remain in care in the private sector but are never notified to the NTP.

### Diagnostic Coverage

The availability of tuberculosis diagnostic tools at different facilities and sectors should be interpreted within the context of the diagnostic pathway planned for the country of interest. For instance, a country may plan for tuberculosis diagnoses to occur primarily in primary healthcare clinics (L1), or the plan may call for patients to be referred to the district hospital (L2) for tuberculosis diagnosis. Given a particular diagnostic approach, one can determine whether there are major gaps in the availability of tuberculosis diagnostic tools at the particular facility level and sector.

### Diagnostic Access at Initial Care Seeking

Diagnostic access is the product of care initiation and diagnostic coverage. If diagnostic access is low, there might be a mismatch between where patients seek care and where diagnostic tools are available. Identifying where this happens can help the program provide more diagnostic capacity in highly accessed facility levels and for preferred provider types.

The diagnostic coverage and diagnostic access indicators provide information on where the greatest gain in case detection can be made. When used at the subnational level, this analysis can identify areas with lower diagnostic coverage or access. Information on diagnostic coverage and access can be combined with information on population size, prevalence or incidence of tuberculosis, and current case notification to identify the priority areas of interventions to improve access to tuberculosis diagnosis. Additional information on feasibility of patient or specimen referral and capabilities in health facilities can help the program select the best way to improve access to tuberculosis diagnostic tools.

### Treatment Coverage

Treatment coverage should be interpreted within the context of the treatment pathway planned for the country of interest. For instance, a country may plan for tuberculosis treatment to be based primarily in primary healthcare clinics (L1), or the plan may call for patients to be referred to the district hospital (L2) for tuberculosis treatment. Given a particular treatment approach, one can determine whether there are major gaps in the availability of tuberculosis treatment at different facility levels and sectors. In addition, one can compare diagnostic coverage to treatment coverage to determine whether there is a major misalignment between where tuberculosis diagnoses are made and where tuberculosis treatment is available. The tuberculosis program should aim to reduce this misalignment to ensure that treatment is available as soon as a tuberculosis diagnosis is made.

### Treatment Access

Treatment access is a product of initial care seeking and treatment coverage. If treatment access is low, there may be a mismatch between where patients initially seek care and where tuberculosis treatment is available. This provides opportunities to examine whether the availability of tuberculosis treatment can be aligned more closely with where patients initially seek care. To do so, it is important to determine whether it is necessary to concurrently improve diagnostic access, given that tuberculosis treatment follows diagnosis. Expanded diagnostic access is often the first step in improving treatment access.

## LIMITATIONS

The PPA methods have several limitations that should be considered when conducting and interpreting the analysis. First, there is a paucity of tuberculosis-specific care-seeking data. Many countries do not have data on care-seeking patterns specific to confirmed tuberculosis cases. In these situations, care seeking for general illnesses is used as a proxy indicator. However, patients who seek care for a general illness may behave differently from those who seek care for tuberculosis or for respiratory symptoms.

Second, even when care-seeking data from representative surveys are available, these surveys are usually performed to provide national estimates. There are generally insufficient data to provide precise estimates at the subnational level. This makes it difficult to generate a subnational PPA.

Third, the diagnostic or treatment coverage in the PPA describes the percentage of facilities with a particular type of tuberculosis services. But these indicators may either underestimate or overestimate the diagnostic or treatment coverage for patients, because the calculation does not account for heterogeneity among health facilities with respect to patient volume, laboratory throughput, or service coverage area.

Fourth, the PPA does not provide important information about delays in getting diagnosis and treatment. Additional studies are needed to describe what happens to patients between the time of initial care seeking and the time of diagnosis, or from the time of diagnosis to the start of treatment. This includes an analysis of time delays, the number of visits required before a patient accesses diagnostic tools or treatment, and patterns of patient referral and sputum specimen transport. Poor quality and inconsistent implementation of tuberculosis services are important contributors to the delay in diagnosis and treatment. The PPA does not provide information on either factor.

Fifth, the PPA does not quantify the frequency of dropout along the different steps of the patient care pathway. Care cascade studies are a complementary way to assess the patient experience. For instance, the care cascade quantifies the proportion of patients lost to follow-up after initially accessing care or receiving a tuberculosis diagnosis.

Finally, the PPA attempts to estimate the likelihood of a patient receiving tuberculosis services on their first visit to a health facility. These access metrics (columns 3 and 5, Figure 2) are based on the coverage of tuberculosis services among health facilities (ie, whether or not the facility has the service in the building). However, there are other factors that are important to ensuring whether a patient will receive appropriate care, even if the tools are available. The quality of the tools, the capacity of the healthcare workers, cost, and the presence of appropriate infrastructure are just a few of the additional factors that will influence whether a patient receives appropriate and timely care. These factors are not captured in the PPA but could be potential areas of expansion of the PPA methods in future research.

## CONCLUSION

The results of a PPA will not in and of themselves transform a tuberculosis program. The results can be considered inputs to a strategic and evidence-based process of identifying, prioritizing and addressing the gaps to patient care. Examples of the use of PPA results for planning are featured in this supplement. In Indonesia and Kenya, subnational PPAs were performed concurrently with WHO-led epidemiological assessments and in advance of external monitoring missions. As such, the results were considered by national tuberculosis programs and their technical partners within the context of a rigorous review of epidemiological trends and a qualitative review of the program’s operations. Kenya also set up county-level meetings of laboratory coordinators and tuberculosis coordinators to discuss and interpret the subnational PPA findings. With the benefit of local knowledge about both the patient population and facility capacities, counties identified localized solutions that were rolled into a revision of the national strategic plan. These models complement data analysis and use should be considered a best practice for robust evidence-based planning going forward. 

## Notes

***Supplement sponsorship.*** This work is part of a supplement sponsored by the Bill and Melinda Gates Foundation.

***Potential conflicts of interest.*** All authors: No reported conflicts of interest. All authors have submitted the ICMJE Form for Disclosure of Potential Conflicts of Interest. Conflicts that the editors consider relevant to the content of the manuscript have been disclosed.
